# Supplementary material for: Fidelity of family centered care model to early disability diagnosis and rehabilitation in the United Arab Emirates
Source: PLoS One. 2024 Apr 4;19(4):e0301094. doi: 10.1371/journal.pone.0301094 (PMC10994312; doi:10.1371/journal.pone.0301094)
Supplement: Attachment — Submitted filename: Review comments for- article PLOS- ONE- FCC.docx [file pone.0301094.s001.docx]

I am greatly happy to review this article **Fidelity of family centered care model to early disability diagnosis and rehabilitation in the United Arab Emirates.**
There are a few points that need to be addressed. The study is good and very helpful for early disability management and making health policies.

1- **Abu Dhabi is the national capital of UAE with an estimated population of about three million - Please give reference.**
2- **10-15% response rate is acceptable in any good study, 150 response is very low.**
3- **How did authors calculated sample size?**
4- **Only 9% of respondent were Emiratis, what is the reason, what is the percentage of Emiratis in Healthcare Professionals?**
5- **43% response were from Physiotherapist, whereas children with disabilities are mostly in close relation with Occupational Therapist, numbers of OT were few in health profession, or what may be the reason?**
6- **What do you mean ‘Place of Training- out-side country response is 77%?**
7- **Authors mentioned that Questioners were translated-and re-translated into Arabic as well, what was the reliability and validity of translation?**
8- **Reference from 1-9 and 10 to onwards there is formatting difference, need**
9- **p-value please write in internationally accepted format in *Italic* (***p***)font in result section.**

**Thanks & Regards.**
