# Supplementary material for: Fidelity of family centered care model to early disability diagnosis and rehabilitation in the United Arab Emirates
Source: PLoS One. 2024 Apr 4;19(4):e0301094. doi: 10.1371/journal.pone.0301094 (PMC10994312; doi:10.1371/journal.pone.0301094)
Supplement: Attachment — Submitted filename: response_to_comments_2.docx [file pone.0301094.s002.docx]

Dear editor

Thanks for considering our paper for publication and we appreciate feedback from the reviewers. As you can see, we have made conscious effort to incorporate feedback from the two reviewers. The changes made are highlighted in colour and below is our point-by-point response to the reviewers’ comments. Thanks once again and looking forward to your feedback.

| **Comments** | **Response** | **Page number** |
| --- | --- | --- |
| I am greatly happy to review this article Fidelity of family centered care model to early disability diagnosis and rehabilitation in the United Arab Emirates. There are a few points that need to be addressed. The study is good and very helpful for early disability management and making health policies. | Thanks for reviewing our paper. |  |
| Abu Dhabi is the national capital of UAE with an estimated population of about three million - Please give reference. | Thanks and we have revised as follow ‘Abu Dhabi is the national capital of UAE with an estimated population of about three million [36].’ | See page 8 |
| 10-15% response rate is acceptable in any good study, 150 response is very low. How did authors calculated sample size? | Thanks for this. We have added how we calculated our sample. Our expected same was 182 which we exceeded with 252 participants. However, there were a number of participants who only completed the demographics. In view of this, we deleted those entries. | See page 8 |
| Only 9% of respondent were Emiratis, what is the reason, what is the percentage of Emiratis in Healthcare Professionals? | We sent the instrument to all potential participants. However, only 252 took part in this study, of which some were deleted due to non-completion. The distribution of Emiratis vs expat health workers was similar what has been reported in the literature. | See page 10 |
| 43% response were from Physiotherapist, whereas children with disabilities are mostly in close relation with Occupational Therapist, numbers of OT were few in health profession, or what may be the reason? | We had a list of all health professionals for this study. you can see, half of them are physiotherapist. Our sample somehow reflect the distribution of allied health professionals in Abi Dhabi. | See page 8 |
| What do you mean ‘Place of Training- out-side country response is 77%? | This refers to country of training. This has been clarified in all the Tables. | See pages 9, 16 and 19 |
| Authors mentioned that Questioners were translated-and re-translated into Arabic as well, what was the reliability and validity of translation? | We did not calculate the reliability. However, we used both internal and external content validity. Internally, the three authors worked separate then reviewed each other’s work. Their work was placed before external reviewers. In our view, the steps appropriate and ensured that the instrument was contextually relevant. | See page 11 |
| Reference from 1-9 and 10 to onwards there is formatting difference, need | Thanks for this. We have formatted both the in-texts and bibliography. |  |
| p-value please write in internationally accepted format in Italic (p)font in result section. | We have italized all the p-value as well as others in the reference. | See pages 13-19 |
|  |  |  |
| The manuscript must describe a technically sound piece of scientific research with data that supports the conclusions. Experiments must have been conducted rigorously, with appropriate controls, replication, and sample sizes. The conclusions must be drawn appropriately based on the data presented. | Thanks for your comments. We have had another look at the manuscript and made some changes in light of your comments. As you can see, we have provided detail information about the study population as well as the sample used in reporting the study. In our view, we used standard procedures for this study and the study findings support our conclusion. |  |
| The authors performed a study addressing “Fidelity of family centered care model to early disability diagnosis and rehabilitation in the United Arab Emirates”. However, there is one unclear area. In "Study participants" on page 7, why were the 252 health care professionals who agreed to participate in the study reduced to 150? Please elaborate on the reason. Also, if necessary, please provide a flowchart | Thanks for this. We have added how we calculated our sample. Our expected same was 182 which we exceeded with 252 participants. However, there were a number of participants who only completed the demographics. In view of this, we deleted those entries. | See page 8 |
